# Tandem NBPF 3mer HORs (Olduvai triplets) in Neanderthal and two novel HOR tandem arrays in human chromosome 1 T2T-CHM13 assembly

**DOI:** 10.1038/s41598-023-41517-3

**Published:** 2023-09-02

**Authors:** Matko Glunčić, Ines Vlahović, Marija Rosandić, Vladimir Paar

**Affiliations:** 1 Faculty of Science, University of Zagreb, 10000 Zagreb, Croatia; 2 Algebra University College, 10000 Zagreb, Croatia; 3 University Hospital Centre Zagreb (Ret.), 10000 Zagreb, Croatia; 4 Croatian Academy of Sciences and Arts, 10000 Zagreb, Croatia

**Keywords:** Evolutionary genetics, Evolutionary theory, Genome informatics

## Abstract

It is known that the ~ 1.6 kb Neuroblastoma BreakPoint Family (NBPF) repeats are human specific and contributing to cognitive capabilities, with increasing frequency in higher order repeat 3mer HORs (Olduvai triplets). From chimpanzee to modern human there is a discontinuous jump from 0 to ~ 50 tandemly organized 3mer HORs. Here we investigate the structure of NBPF 3mer HORs in the Neanderthal genome assembly of Pääbo et al., comparing it to the results obtained for human hg38.p14 chromosome 1. Our findings reveal corresponding NBPF 3mer HOR arrays in Neanderthals with slightly different monomer structures and numbers of HOR copies compared to humans. Additionally, we compute the NBPF 3mer HOR pattern for the complete telomere-to-telomere human genome assembly (T2T-CHM13) by Miga et al., identifying two novel tandem arrays of NBPF 3mer HOR repeats with 5 and 9 NBPF 3mer HOR copies. We hypothesize that these arrays correspond to novel NBPF genes (here referred to as NBPFA1 and NBPFA2). Further improving the quality of the Neanderthal genome using T2T-CHM13 as a reference would be of great interest in determining the presence of such distant novel NBPF genes in the Neanderthal genome and enhancing our understanding of human evolution.

## Introduction

### High-quality Neanderthal genome sequence

Impressive progress by Pääbo et al. in high-quality sequencing of Neanderthal’s genome has opened new avenues in studying relation of modern humans and our closest extinct relatives Neanderthals, in quest of searching “what makes us human”^1–6^. The high-quality genomes *Denisova 5 AltaiNea.hg19*^7,8^, Chagyrskaya 8^6^, and *Vindija 33.19*^5^ were determined. Under the assumption that Neanderthals had the same mutation rate (1.45× mutations per generation per base pair)^9^ and generation time as for present-day humans (29 years), it was suggested that Chagyrskaya 8 lived ~ 30 ky after Denisova 5, and ~ 30 ky before Vindija 33.19^6^. An analysis of these high-quality genomes revealed significant changes in genes expressed in the striatum of the brain, indicating the potential evolution of unique functions in the Neanderthal brain^6^.

### Human specific ~ 1.6 kb tandem repeat units in NBPF genes

Neuroblastoma is a solid malignancy that primarily affect children and has been the focus of intense research^10,11^. The NBPF gene family was originally identified by the disruption of one of its members in a neuroblastoma patient^12^. The NBPF genes are located on human chromosome 1 and contain a repetitive structure of ~ 1.6 kb tandem repeat units known as Olduvai domains (also called NBPF domains, NBPF repeats, or DUF1220 domains), which code for Olduvai protein domains (previously called DUF1220)^13–19^ involved in human brain evolution. The term Olduvai for these repeat structures is referred to as Sikela–van Roy terminology^19^. Alternatively, in accordance with Willard’s terminology used for tandem repeats in centromeric region of human genome^20–22^, the repeat units in NBPF sequences were referred to as NBPF monomers^23^.

Studies have found that the copy number of Olduvai domains is correlated with various aspects of brain function and pathology, including brain size, cortical neuron number, IQ scores, cognitive aptitude, autism, schizophrenia, microcephaly, macrocephaly, and neuroblastoma^13,15,18,24–33^. The association between HLS Olduvai domain copy number and the human brain evolution with increased cognitive function was suggested by Sikela et al.^15,19^.

Interestingly, the copy number of NBPF domains in nonhuman species generally decreases with increasing phylogenetic distance from humans, with humans having the highest number (~ 300 copies) followed by great apes (~ 38–97 copies), monkeys (~ 48–75 copies), and non-primate mammals (~ 1–8 copies), while these domains are mostly absent in non-mammalian species^15,17,18,27,29,34,35^.

In our previous research, we found that the human specificity of NBPF copy number variation is significantly more pronounced in tandemly organized NBPF 3mer higher order repeats (HORs) than in individual NBPF ~ 1.6 kbp monomer copies^23^. Specifically, we observed a copy number of 47 HORs in humans, whereas chimpanzees, gorillas, orangutans, and rhesus macaques showed zero copy number. Recent computations using higher quality sequencing, specifically the hg38.p14 human reference genome and NC_036879.1 chimpanzee ensemble, have yielded similar results^36^. Based on these findings, we have hypothesized that the tandemly organized ~ 4.8 kb NBPF 3mer HOR copy number may provide an additional evolutionary signature, in conjunction with the individual ~ 1.6 kb NBPF primary repeat/Olduvai domain copy number effect, potentially leading to a coherent overall effect.

In this study, we aim to compare the copy number of NBPF tandemly repeated HORs in Neanderthals and humans, as well as in comparison to the human complete T2T-CHM13 assembly^37–39^ and chimpanzee reference Pan troglodytes NHGRI_mPanTro3-v1.1-hic.freeze_pri (CM054434.1 Pan troglodytes isolate AG18354 chromosome 1, whole genome shotgun sequence). Our findings could shed light on the role of NBPF genes in human evolution, as well as on the genetic differences between Neanderthals and modern humans.

### The ~ 171 bp alpha satellite monomers and alpha satellite nmer higher order repeats (HORs) in centromeres of human genomes: a HOR prototype

Most pronounced tandem repeats in human genome are alpha satellites located in the centromeric regions of chromosomes. These repeats consist of ~ 30–50% diverged ~ 171 bp alpha satellite monomer units, which serve as the primary repeats^40,41^. Frequently, these alpha satellites are organized into higher order repeats (HORs)^22^. HORs are composed of monomers arranged in multimeric repeat HOR copies that are tandemly positioned. The level of divergence between HOR copies is very small, often less than 5%, which is an order of magnitude smaller than the divergence observed between neighboring monomers^21,22,37,38,41–49^. These primary repeats and tandem HOR repeats are commonly referred to as Willard’s terminology. The repetitive structure of alpha satellite repeats and the organization of HOR arrays have significant implications in the study of chromosome biology, genome instability, evolution, and human disease. They serve as a source of genetic and epigenetic variation, contributing to the dynamic nature of the genome^38,50^.

### NBPF 3mer HORS/Olduvai triplets

In 2011, we applied our robust HOR-searching algorithm, GRM (Global Repeat Map algorithm), to the Build 36.3 human genome assembly for chromosome 1, which led to the discovery of tandemly organized ~ 4.8-kb 3mer HOR copies in NBPF genes^23^. The GRM algorithm revealed that each 3mer HOR copy is composed of three ~ 1.6 kb NBPF monomers, denoted m1, m2 and m3, respectively^23^.

**Figure 1:**
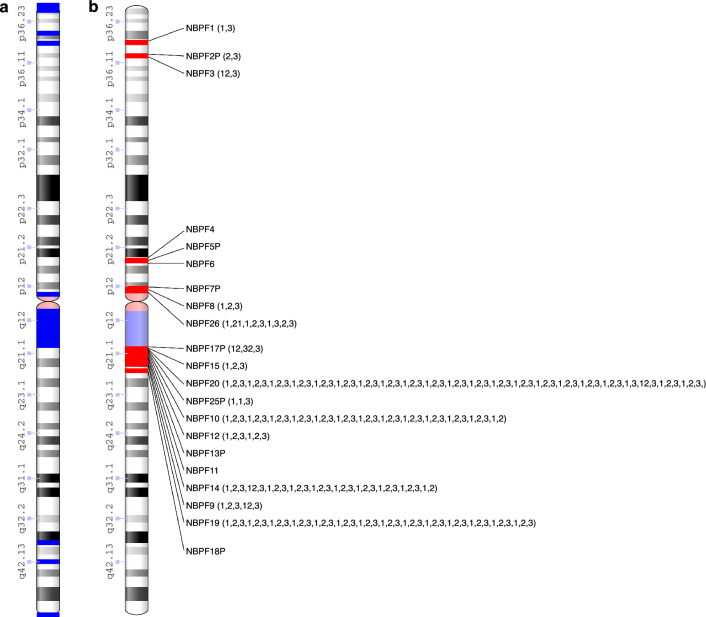
Maps of gaps, NBPF gene members, and their content of Olduvai repeats in the human chromosome 1 genome hg38 assembly. (**a**) Positions of gaps (blue rectangles) (indicated in the assembly as ‘N’s) larger than 2000 bp. (**b**) Positions of NBPF gene members (red rectangles) (according to the National Center for Biotechnology Information) and arrangement of Olduvai domain subtypes^51^. Only HLS1 (1), HLS2 (2), and HLS3 (3) Olduvai domains subtypes are indicated. Domains consisting of two different HLS types are denoted with two connected numbers (e.g., the second domain in NBPF26 consists of half HLS2 and half HLS1, is indicated as 21). It is evident that the hg38 assembly has a gap at the position of the NBPF1 and NBPF7P genes.

In 2012, the same pattern was recognized through another method, which involved analyzing the similarity between the ~ 1.6 kb Olduvai domains present in NBPF genes (Fig. 1). This analysis revealed that the ~ 1.6 kb Olduvai domains are predominantly organized in repeating triplets with minimal divergence between the triplets^16,17^. Previously referred to as HLS/DUF1220 triplets in Sikela–van Roy terminology, and consist of three domains designated as HLS1, HLS2, and HLS3, these triplets have recently been renamed Olduvai triplets^19^. Table 1 presents the correspondence between different names for the same repeat pattern obtained through different computational methods.

**Table 1:** Correspondence of Sikela-van Roy terminology and Willard terminology for NBPF repeats.

Sikela-van Roy terminology	↔	Willard terminology
DUF1220 domains/Olduvai domains	↔	NBPF monomers
HLS-1	↔	m1
HLS-2	↔	m2
HLS-3	↔	m3
DUF1220 triplets/Olduvai triplets	↔	NBPF 3mer HOR copies

Willard terminology was used generally for *n* monomers in *n*mer HOR copies with *n* distinct monomers of types m1, m2, … m*n*^21,22^. For the NBPF repeats, Willard terminology is applied here to the special case of n = 3, with three types of ~ 1.6 kb NBPF monomers m1, m2, and m3. In the computational method for HOR identification, Willard terminology is characterized by identification of ~ 4.8 kb NBPF 3mer HOR copies (i.e., Olduvai triplets) in the first step of computation, and their constituent monomers m1, m2 and m3 are identified in the second step within HORs^23^. On the other hand, Sikela–van Roy terminology is characterized by identification of Olduvai domains (i.e., 3mer NBPF monomers) in the first step of computation, and their corresponding Olduvai triplets (i.e., 3mer HOR copies) identified on the basis of divergence among Olduvai domains.

Additionally, it is worth noting that, besides the NBPF 3mer HOR, the same HOR-searching GRM computation also identified two others prominent HORs in human chromosome 1^23^. In hornerin genes, novel quartic HORs were discovered, consisting of primary, secondary, tertiary, and quartic repeats with lengths of approximately ~ 39 bp, ~ 0.35 kb, ~ 0.7 kb, and ~ 1.4 kb, respectively^23^.

Moreover, the pronounced well known centromeric HOR pattern in the centromeric region of chromosome 1 is the canonical alpha satellite 11mer HOR^20,44,46,52^ with suprachromosomal assignment SF2^52^. Recently, a canonical 6mer HOR with suprachromosomal assignment SF1 was identified^37,48^. Here, using the GRM algorithm both the 11mer and 6mer canonical HORs were identified in hg38. In the GRM result, the divergence between HOR copies in 11mer HOR array is a few times smaller than between 6mer copies, corresponding to homogene and divergent HORs, respectively.

## Results and discussion

### Exclusively human-specific GRM diagrams

**Figure 2:**
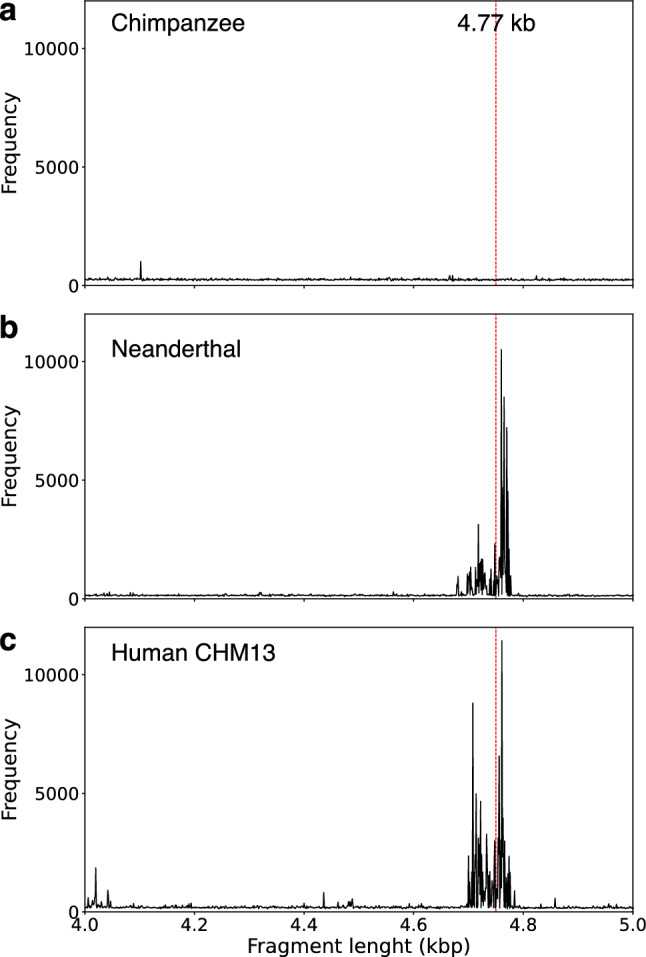
GRM diagrams for 140–150 Mb segment of chromosome 1: (**a**) Chimpanzee *NHGRI_mPanTro3-v1.1*; (**b**) Neanderthal AltaiNea.hg19, and (**c**) Complete human T2T CHM13 assembly. GRM diagrams have pronounced GRM peaks at ~ 4.8 kb for Neanderthal and human genomes, while for chimpanzee the peak at ~ 4.8 kb is absent.

In the first step, the GRM diagrams for chromosome 1 in Neanderthal’s AltaiNea.hg19 assembly^4^ and in recent complete human T2T assembly T2T-CHM13^37–39,53^ are computed (Fig. 2b,c, respectively). These results are then compared to GRM diagrams for hg38.p14 (NC_000001.11) assembly of the human chromosome 1 and other nonhuman assemblies from Ref.^36^, namely the chimpanzee assembly Clint_PTRv2 (RefSeq sequence NC_036879.1) (Fig. 2a), gorilla assembly Kamilah_GGO_v0 (RefSeq sequence NC_044602.1), orangutan assembly Susie_PABv2 (RefSeq sequence NC_036903.1) and rhesus macaque assembly Mmul_10 (RefSeq sequence NC_041754.1). The GRM peak at ~ 4.8 kb corresponds to the ~ 4.8 kb tandemly organized canonical NBPF 3mer HOR copies (m1m2m3), which are based on the ~ 1.6 kb NBPF monomers of types m1, m2, and m3. Consensus NBPF monomers m1, m2 and m3 for Neanderthal, hg38 human and T2T-CHM13 human assemblies are given in the Supplementary Table 1.

### NBPF 3mer HOR copy aligned schemes

**Figure 3:**
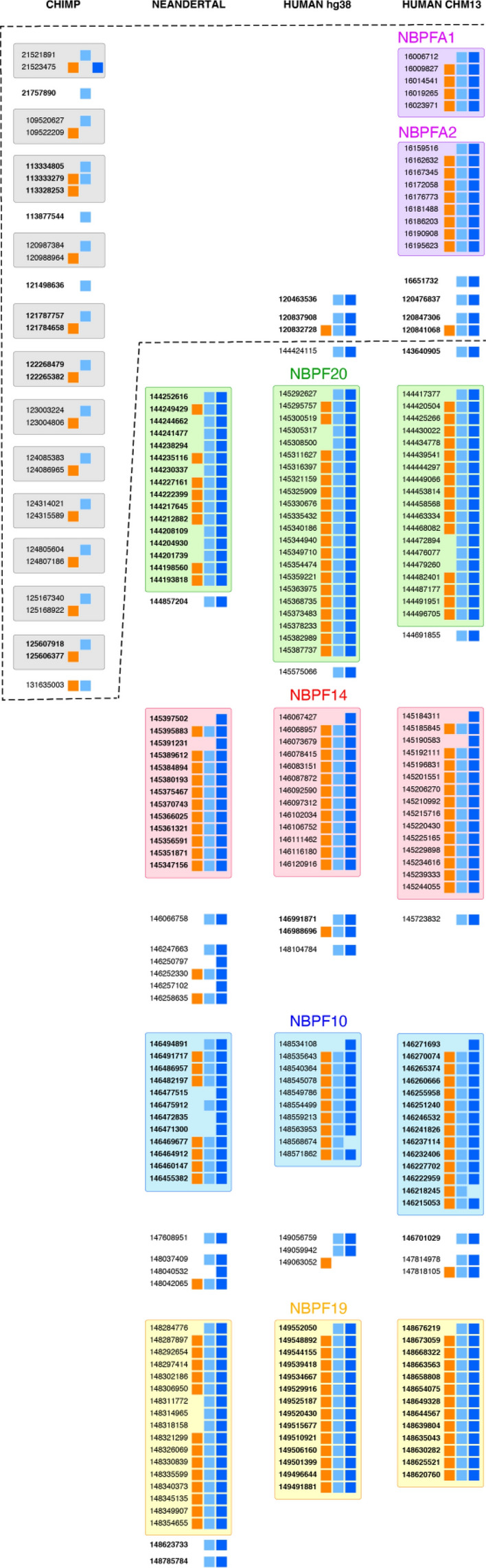
Comparison of NBPF monomer alignment scheme for NBPF 3mer HOR copies for chromosome 1 in chimpanzee *NHGRI_mPanTro3-v1.1* (1st panel), *Neanderthal AltaiNea.hg19* (2nd panel), *human hg38.p14* (3rd panel) and complete *human T2T-CHM13* (4th panel). Boxes represent three types of NBPF monomers, denoted m1 (orange), m2 (light blue) and m3 (blue). Each row of boxes (three in canonical HOR copy, two or one in variant HOR copies) represents an NBPF HOR copy. In front of each row (HOR copy) its start position in the genomic sequence is given. Initial positions that are in bold indicate monomers that appear in the original sequence in the reverse-complement orientation. Each array of tandemly arranged HOR copies is separated from neighboring arrays and/or isolated individual monomers by blank space. Consensus monomers determined by GRM algorithm for human and Neanderthal genome are almost the same (average divergence less than 4%). A characteristics of chimpanzee NHGRI*_mPanTro3-v1.1* assembly is the absence of canonical HOR copies. Moreover, except in the second variant HOR copy m1m3 (at position 21,523,475 bp), the m3 NBPF monomer is missing. A pronounced additional segment, identified only for the T2T-CHM13 assembly, is the appearance of two novel tandem arrays of NBPF 3mer HOR copies, the 5-copy and 9-copy arrays, interpreted as belonging to the novel genes named NBPFA1 and NBPFA2 genes.

Figure 3 compares the Global Repeat Map (GRM) results for aligned NBPF 3mer higher order repeat (HOR) copies, including canonical and variant copies, in chromosome 1 across different genome assemblies. The panels show the results for the chimpanzee NHGRI_mPanTro3-v1.1 assembly (1st panel), Neanderthal AltaiNea.hg19 assembly (2nd panel), human hg38.p14 assembly (3rd panel), and human complete T2T-CHM13 assembly (4th panel). To construct these aligned NBPF HOR schemes, we first present the aligned NBPF 3mer HOR copies in the human hg38.p14 assembly (3rd panel), which are similar to previous results computed for HLS domains^16^ and GRM results^23,36^. Some small differences are due to variations in the computational methods used to identify primary repeats of ~ 1.6 kb and/or ~ 4.8 kb secondary HOR repeats, as well as the quality of the sequenced genome. In this presentation, the NBPF monomers are horizontally grouped into HOR copies (i.e., within each 3mer HOR copy—canonical or variant), which are then aligned vertically. A blank space is inserted between any two neighboring groups of tandemly organized HOR copies, treating individual isolated monomers of types m1, m2 or m3 as single-monomer HOR copies.

### Human hg38.p14 panel

The human hg38.p14 (3rd panel) is characterized by presence of four pronounced tandemly organized arrays of NBPF 3mer HOR copies (canonical and variant). These arrays include a 22-copy array, 13-copy array, 10-copy array, and 14-copy array, respectively, (Table 2) with at least two neighboring canonical HOR copies arranged in tandem (Fig. 3). These arrays correspond to the four prominent NBPF genes found on human chromosome 1: NBPF20, NBPF14, NBPF10, and NBPF19, respectively. Combined, these four NBPF genes encompass a total of 59 NBPF 3mer HOR copies (52 of which are canonical). It is important to mention that the order of these four tandem arrays is reversed compared to the ordering specified in Ref.^16^. To facilitate visual clarity, each tandem array is color-coded: green, red, blue, and orange, respectively. Beyond these four HOR copy arrays, there are an additional seven smaller groups of scattered HOR copies. Three of these scattered groups are positioned above the 22-copy array corresponding to the NBPF20 gene.

**Table 2:** Number NBPF 3mer HOR copies in tandemly organized 3mer HOR copies (canonical and variant) in chromosome 1 of Neanderthal AltaiNea.hg19, human hg38.p14 and complete human T2T-CHM13 assemblies (deduced from Fig. 3).

Assembly	No. of canonical and variant HORs gene NBPF						Total no. of canonical HORs
	20	14	10	19	A1	A2	
Chimpanzee NHGRI_mPanTro3-v1.1							0
Neanderthal AltaiNea.hg19	16	13	12	17			39
Human hg38.p14	22	13	8	13			52
Human T2T-CHM13	19	15	14	13	5	9	64

### Horizontal alignment of 2nd, 3rd and 4th panels

In the next step, we adjusted the relative positions of the four prominent NBPF 3mer HOR tandems in panels 2, 3, and 4 of Fig. 3. This was done by aligning the first rows of each NBPF gene horizontally while maintaining the distances within the tandem arrays. By employing this spreading technique, the first rows corresponding to the NBPF20 gene in Neanderthal AltaiNea.hg19, human hg38.p14, and human T2T-CHM13 were horizontally aligned. Similarly, the other three notable NBPF genes (NBPF14, NBPF10, and NBPF19) were horizontally aligned as well. As a result of this spreading based on the positions of the four significant NBPF genes within each panel, the top sections of the hg38.p14 (3rd panel) and CHM13 (4th panel) are positioned above the NBPF20 gene (green area). The top section of the 3rd panel (for hg38.p14) exhibits three distinct sets of HOR copies (2-monomer variant HOR copy, canonical HOR copy + 2-monomer variant HOR copy, 2-monomer variant HOR copy).

### Neanderthal AltaiNea.hg19 panel

The Neanderthal AltaiNea.hg19 (2nd panel) exhibits four distinct and tandemly organized arrays of NBPF 3mer HOR copies (canonical plus variant) in a top-to-bottom manner: 16-copy, 13-copy, 12-copy, and 17-copy, respectively (Fig. 3, Table 2). These arrays correspond to four prominent NBPF genes located in human chromosome 1: NBPF20, NBPF14, NBPF10, and NBPF19. Altogether, these four NBPF genes contribute to a total of 58 NBPF 3mer HOR copies (39 canonical).

When comparing the alignment of NBPF 3mer HORs between Neanderthal AltaiNea.hg19 and human hg38.p14, the tandem HOR groups from the corresponding NBPF genes appear to have a similar order. However, it is important to consider that the Neanderthal genome was sequenced based on a comparison with an earlier human assembly, and despite its relatively high-quality sequencing, there were still gaps in the genome sequences. In Fig. 1a the positions of sequence gaps discussed in the region of NBPF genes are presented. Notably, human hg38.p14 has a sequencing gap of 18 Mb, which is located in proximity to the position of the NBPF20 gene. Additionally, there are several other sequencing gaps of approximately 50 kb that are further away from the NBPF genes. In the Neanderthal AltaiNea.hg19 assembly, there is a 21 Mb gap near the positions of NBPF genes, along with several other gaps of around 150 kb, 100 kb, and 50 kb close to the region of NBPF genes. Consequently, while comparing the results between Neanderthal AltaiNea.hg19 and human hg38.p14 is reasonable, it is possible that some HOR copies have been missed in the sequencing process due to the presence of more gaps in the region near the four 3mer HOR copy-rich arrays in Neanderthal.

### Human T2T-CHM13 panel

The complete human T2T-CHM13 assembly, represented in the 4th panel, displays four prominent and tandemly organized NBPF 3mer HOR copies that roughly align with the pattern observed in hg38.p14 (3rd panel): a 19-copy array (green area), a 15-copy array (red), a 14-copy array (blue), and a 13-copy array (orange) (Fig. 3, Table 2). This ordering of tandemly organized NBPF HOR-copy arrays roughly corresponds to the NBPF genes 20, 13, 10 and 19, respectively, similar to the hg38.p14 case. Additionally, we identified eight scattered small groups of HOR copies outside the four major HOR copy arrays in the T2T-CHM13 sequence. Notably, above the 19-copy array (green area), there are three small, scattered groups exhibiting the same HOR pattern as observed in hg38.p14 (2-monomer variant HOR copy, canonical HOR copy + 2-monomer variant HOR copy, 2-monomer variant HOR copy). However, at the top of the 4th panel, two additional tandemly organized arrays of NBPF 3mer HOR copies are present: a 5-mer array and a 9-mer array (violet area). We tentatively assign these NBPF tandem arrays to two genes, designated as NBPFA1 and NBPFA2, respectively. These arrays are positioned relatively close to the telomeric region, located more than 100 Mb away from the known NBPF genes. The total number of constituting NBPF 3mer HOR copies (canonical plus variant) in the T2T-CHM13 assembly is 75 (64 canonical). All NBPF genes with tandemly organized Olduvai triplets (canonical NBPF 3mer HOR copies) are located in the 1q region^16^. However, several NBPF genes are also known to be located also in the 1p region, but they lack tandemly organized Olduvai triplets. For instance, in the hg38.p14 assembly, the gene NBPF1 in 1p36.13 contains no Olduvai triplet, while the gene NBPF8 in 1p11.2 contains only one Olduvai triplet^16^. The two novel NBPF genes with tandemly organized Olduvai triplets, discovered here in the CHM13 assembly and absent in hg38.p14, are referred to as NBPFA1 and NBPFA2. As the Neanderthal AltaiNea.hg19 assembly was sequenced based on a comparison to the previous human reference genome similar to hg38.p14, it is expected that these two novel NBPF genes are also absent in the Neanderthal AltaiNea.hg19 assembly.

## Conclusion

It is shown that the abundant tandemly organized NBPF 3mer HOR copies in complete human assembly T2T-CHM13 and in Neanderthal AltaiNea.hg19 assembly of chromosome 1 are exclusively human (homo sapiens and Neanderthal) specific, because they are completely absent in chimpanzee chromosome 1. In addition, we identified two new tandemly organized arrays of NBPF 3mer HOR copies in the complete human T2T-CHM13 assembly which we assign to two new genes. On the other hand, Sikela and collaborators have shown that the number of NBPF repeats, which is only about twice as high in humans compared to chimpanzees, correlates with the gradual increase in primate cognitive abilities^24,26,27^. We hypothesize that the increase of cognitive abilities is coherently increased by tandemly organized NBPF higher order repeats (HORs) which are highly present in human and Neanderthal genomes and absent in the chimpanzee genome. In conclusion, our findings highlight the need for an additional sequencing of the Neanderthal genome, utilizing the T2T-CHM13 human genome as a reference. This proposed approach holds the potential to provide more accurate and detailed information, which would play a crucial role in advancing our understanding of the evolution of cognitive abilities in both human and Neanderthal populations. Particularly intriguing is the contrast with chimpanzees and other primates, as they entirely lack tandemly organized NBPF HORs, further underscoring the significance of conducting comparative genomic analysis. By shedding light on the genetic factors that contributed to cognitive development in our evolutionary history, this research could offer valuable insights into the shared and distinct traits between humans and Neanderthals. Ultimately, such knowledge may enrich our comprehension of the complexities of human evolution and contribute to broader discussions in the field of evolutionary biology.

It is interesting to note that a sophisticated phenomenon of cognitive development is related to the underlying tandem HOR pattern, which is based on the concept of DNA symmetries. This pattern represents an evolutionary trajectory characterized by symmetries, resulting in increased order and a reduction in information entropy.^36,54,55^.

## Materials and methods

The NBPF HORs were identified in the NCBI assembly (2023) of human, pan troglodytes and Neanderthal genomes using the GRM algorithm^56–58^. The GRM algorithm is an efficient and robust method specifically designed to detect and analyze very large repeat units, such as HORs, within genomic sequences. It effectively reduces computational noise associated with detecting longer and more complex HOR repeat units, ensuring the accurate identification of peaks corresponding to HOR copies. Unlike other methods, the GRM approach directly maps symbolic DNA sequences into the frequency domain using a complete K-string ensemble, avoiding the need for statistical adjustments and local optimization of individual K-strings. This unique feature allows for straightforward identification of DNA repeats in the frequency domain without the need for mapping symbolic DNA sequences to numerical sequences. The GRM algorithm demonstrates robustness in handling deviations from ideal repeats, making it suitable for repeats with substitutions, insertions, and deletions. Additionally, it provides parameter-free identification of repeats, enabling the determination of consensus lengths and consensus sequences for primary repeats and HORs. The GRM method generates a global repeat map in a GRM diagram, identifying all prominent repeats in a given sequence without any prior knowledge of the repeats. Furthermore, once the consensus repeat unit is determined using GRM, it can be further combined with a search for dispersed HOR copies or individual constituting monomers.

Specifically, NBPF HORs in this study were identified through the following steps:

(i) Using GRMapp (the GRM graphical user interface application is freely available at http://genom.hazu.hr/tools.html), NBPF monomers were identified within the entire human hg38, human CHM13, Neandertal and chimpanzee chromosome 1 assemblies. GRMapp provides all tandem repeats (TRs) in the analyzed assembly as its output. From the list of all TRs, those with lengths of ~ 1.6 kb in the region of the NBPF genes were selected and subjected to GRM diagram analysis within GRMapp. To be classified as NBPF monomers, the GRM diagram must exhibit peaks at ~ 1.6 kb and multiples at ~ 3.2 kb and ~ 4.8 kb, indicating the existence of higher-order structures (HORs).
(ii) The extracted NBPF monomers were compared to each other, and a divergence matrix was created. From the divergence matrix, monomer families were identified, encompassing all monomers that differ from each other by less than 5%. In this manner, three monomer families, m1, m2, and m3, were obtained.
(iii) For each monomer family, a consensus sequence was generated using the stand-alone tool for multiple-sequence alignment, pyabPOA (pyabpoa 1.0.0a0), available at https://github.com/yangao07/abpoa. The consensus sequences for m1, m2, and m3 are provided in Supplementary Table 1.
(iv) Each chromosome 1 assembly (chimp, Neanderthal, human hg38, and human T2T-CHM13) was searched with all three consensus sequences, m1, m2, and m3, using the Edlib open-source C/C++ library for exact pairwise sequence alignment^59^. The search was conducted base by base for the entire chromosome, considering both the direct and reverse complement consensus sequences. The resulting tables for each organism are provided in Supplementary Table 2.
(v) The results of the search in step (iv) were presented graphically (Fig. 3) in a way that all monomers of the same family (m1, m2, m3) are located in the same column and colored with the same color.

### Supplementary Information

Supplementary Table 1.

Supplementary Table 2.

## Data Availability

All genomic sequences are freely available at the National Center for Biotechnology Information (NCBI) website https://www.ncbi.nlm.nih.gov. The GRM graphical user interface application (JAR file) is freely available at our project’s website http://genom.hazu.hr/tools.html. It can be run on any platform which have Java Runtime Environment (JRE) installed (freely available at https://www.oracle.com/java/technologies/javase-downloads.html).
