# Supplementary material for: Tandem NBPF 3mer HORs (Olduvai triplets) in Neanderthal and two novel HOR tandem arrays in human chromosome 1 T2T-CHM13 assembly
Source: Sci Rep. 2023 Sep 2;13:14420. doi: 10.1038/s41598-023-41517-3 (PMC10475015; doi:10.1038/s41598-023-41517-3)
Supplement: Supplementary file 1 — Supplementary Table 1. [file 41598_2023_41517_MOESM1_ESM.docx]

Supplementary table 1. Consensus sequences of NBPF monomers

m1 NBPF consensus

CCTGAAGGCTGGTCATGATAGAAATTCCTCGGTTTTTCTCCCAGAAACTGTGGGTAAAATGTCCCTATTCTAGTAGATCGTTATCCCAATATCATTTGTCCCAAGTTTGTGCAAACAGTTATGCCATATTTTTCCAATCAACTTAAAGCAAATACCCTCAAATGATTTCTAGGAGAAAAACTGCAATATTTAGCCCTGTCTCATCAAATACTCAGATTGTTCATGGTTGTGAGGACTTTAGACACTGAAATTAGAGTGAAAAAGGAAATCTACAAACCCTTGAGTCAAAATCATAGTTCTCTGAATTTGTCACATCTGCCCAGGTCCAATGTCATGAGAATAGGATCAGGGCGCCACAGGTATGGCCTGAGACTAGGAAGAGAGTCTTGCTCACTGACCCATCCCTTGTCTGGGCTTCCAGGTAGAACTAGAGTTTCATTCAACCTACATGTGCCTATAGGTCCTCCCTGTGGCAATGACATCTCTCAGCTCAGTAATGGCCACTTGGAGCAGGAATATGATCTTTATATGGAAGACTCAGTGGATCCTTATCACCTTCATAGAAAGGTACTCACCTCCCACGTCAAGAGAAAAGCCAACATGTTTTTCCTCCAATGCATAAAAGGAACTTCCATAGGGCTGGCAGGAGTCAGGCTGTTCAAGACAACTGGAAGGAGTTGAATAACATCTATCCAGTGAGTCCTGCAAGACTTCAGGCTCTACTACCTCCAGCAGCTCCCTGCTGAGCCTGGAAAAGGAGGAAAAAGTAAAGAATAAGCCAGGGGAAATCAGACACAACAGAGCCCCAACTAGGTTTCATGGGTAGCATAGGGAAGTGGTTAAAAAACTAAAAGGATAGATCCATTAATGAGGTAACAAATTATTGCCTTCATGTTGGGACAGAACAGGGCCAAATGGAAAAGAATGAAAGAGAAAGACAGATAGACACACACACACACACACACACACACAACACACACACACACACACACAGAGAGAGAGAGAACGAGCTCAGTGAATTGTCCAGGTGACACACTGATGAGGGAGTAACAGGACACTCTGAGTTAGTGCCCTCAGGACACACAGCATACAGGGATCATGAAAAGACTGTGCTCAATAATTTTCCATAAAATGTGCTCAAGTTTCCATGCAGTCGCCATGAGAATACAGTTTTTGAAGTCTGGTCCACCTACAGTAGGTTAGTAAATGATAAGGGGAGGAAGAAATGGAAACCTAAATATCTACTGCAATGAAAACCAACAGCAATGTTAGTAGGAATAATTCAGGCTTGCTTGAAAAGATGTAATCGATAATGTCAGCCCGCTCTGTTTTCCCTGAACCAGGAGTCTCCAGATGTCAACACAGAAGTAGCTGTTCACAATTGCTCAGTTACCTGGGGCATGGTGGGCCTTGGTCTTCTTCCTCTTCTTGGTCCTTTTTAATTCCTGCAATACATTCAGACAGGGACAGACAAAATAAGCCAATTCACCTACACCCATAACAGTCCACTGTCTAATCCCCACACAGGGATCTCAGGCTCCTCAGCATGAGAACAGGACAATGTGAGAGATATACTTCAGGAGG

m2 NBPF consensus

CCTGAAAGCTGGTCATGATATTCTTTGGTTTGCATCTCAGAACCAAGGGTGAAATATCCCCATTCTGGTAGATCGTTATCCCAAAATCATTTATCCCAAGTTTGTGCAAACAGTTATGCCTTATTGTTCCCATCAGTTCAAAGAAAATGCCCCAGATGATTTCTAGGAGGAAAACTGCAGTATTCAGCCCTGTCTCATCAAATGCCCAGCTCGTTCATGGATGCAAGAATTTTAGACACTGAAATTAGAATGAAGGAGGAAATCTACAAACCCTTGAGTCCAAATCATACTTCTGTGAATTTTTTACATCTGCCTGGGTCCAATGTGCTGAGAGCGGGCTCAGGTTGCCACAGGCATGGCTGGAGACTAGGAATAGAGCCTTGCTCACTGACCCATTTCATGTCTAGGCTTCCAACTGAGACTACAGTTTCATTACAACCTATATGCGCCCATAGGTCCTGCCTGCGGCAATGACATCTCTCGGGTCAGTAAGGGCCACTTGGAACAGGAATATCACCCCTATCTGGAAGACCAGGTGGAGGCTTATCACCTTCACAGTAAGGTACTCACTGTCCACGTCAAGAGCCAAGCCAAGGTACTGTTCCTCCAATGAGTAAACAGCACTGCTGTAGGGCTGGCCTAAGTCAGGCAGTTCAAGATAACCTGAAGGAGTCGAATAACATCTATCCAGTGAGTCCTGCAAGACTTCAGGCTCTTTCTCATCCAGCAGCTCCCTGCTGAGCCTGGAAAAGTAGGAAAAAGTAAAGAATAAGCCAGGGGGAATCAGAAACCACACAGCCCCAGCTAGATTTCATGGCTAACATAAGGAACTGTTTAAAAAGAAAAAGGACAGATCCATTAATGAGGTAATGAATTATTGCCTTTATGTTGGGATAGACCAGGGCCAGGTAGAAAAGAATGAAAGAGAAAGACAGGGAGAGGGAGGAGAGAGAGAGAGAGGAGAAAGTGAGCTCAGCGAATTGGCCGGGTGACACACTGATGAAGGGGTCAAAGGACACTCTGAGTTAGTGCCCTCGGGACACACAGCGAACAGTGATCATGAAAAGAGTGGGCTCAATAATTTTCCATAAACTTGCTCAAGATTCCATGCAGTTGCCATACAGCCTTTGAGGTATGGTCAACCTATAGTAAGTTAGTAAATGATAAGGGGAGGAAGAAATGGAAACCTAAACATCTACTGCAATGAAAACCAACAGCAATGTCAGTAGGAGTAATTCAACCTTCGTTGAAAACATGAAATTGAACACACTCTTGTTTTCCCTGGACCTGGCATCTCCAGGTGTCAACACAGAATTAAGCATCCATAATTGCTCAAAGTTACCTGGGGCATGATGGGTCTTGGTCTTCTTCCACTTCTTGGTACTTTTCAATTTCTGCAATAAGTTCAGACATGGACAGACATATTAAGCTGGTTCTCCTACACACATAACAATCCACTGTCTAATCCTCACACAGGGACTTCAGGCTCCTCAGCATGAGAATAGGACACTGTGAGAGATAGTCTTCAGGAGGCCTGAAGG

m3 NBPF consensus

CCTGAAGGCTGATCACCATAGAGATTCCTTGGTTTTTGTCCCAGAAACTGTGGGTAAAATTCCCTATTCTGGTAGATCGTTATCCCAATATCATTTGTCCCAAGTTTGTGCAAATGGTTATGCCATATTTTTCCAATCGATTTAAAGCAAATGCCCCCAAATGGTTGCTAGGAGAAAAACTGCACTATTCAGCCCTGTCTCATCAAATACTCAGATTGTTCATGGTAGCGAGGATTTTAGACGCTGAAATTAGAGTGAAGGATGAAATCTACAAGATCTACAAAATTGAGACAAAATCAGAGTTGTGTGAATTTGTCACATCTGCCCAGGTCCAACGTCATGAGAGTAGGATTAGGGCGCCACAGGCATGGCCTGAGACTAGGAAGAGAGCCTTGCTCACTGACCCATCCCTTGTCTGGGCTTCCAAGTGGAACTAGAGTTTCATTCAACCTACATGTGCCTATAGGTCCTCCCTGTGGCAATGACATCTCTCAGCTCAGTAAGGGCCACTTGCAGTAGGAATATGACCCTAACCAGAAGACTCAGTGGATCCTTATCACCTTCATAGAAAGGTACTCACCATCCATGTCAACAGCCAAGCCAACACGCTGTTGCTCCAATACGTAAAAGGCACTTCTGTAGGGCTGGCATGAGTCAGTCAGTTCAAGACAACCTGAAGGAGTTGAATAACATCTATCCAGTGAGTCCTGCAAGACTTCAGGCCCTTTCTCATCCAGCAGCTCCCTGCTGAGCCTGGAAAAGTGGGAAAAAGTAAAGAATAAGCCAGGGGGAATCAGAAACCACACAGCCCCAGCTAGATTTCATGGCTAACGTAAGGAAGAGTTTGAAAAGAAAAAGGACAGATCCATTAATGAGGTAACAAATTATTGCCTTTATGTTGGGATAGAACAGGGCCAGGTAGAAAACAATGAAAGAGAAAGACAGACAGAGAGAGACAAAGAAGAGACAGAGACAGAGACAGAGAGAAAGTGACCTAGTGAATTGGCCAGGTGACATACTGGTAAGGGAGTCAAAGGACACTCTGAGTTAGTGCCCTCATGACACACAGCAAACTGTGATCATGAAAAGAGTGAGCTCAATAGTTTTCCATAAAATATGCTCAAAATTCGATGCAGTGGCCATGAGAGTACAGCTTTTGAAGTATGGTCAACCTATGGTACGTTAGGAAATGATAAGGGGAGGAAGAAATGGAAACCTAAACATCTACTGCAATGAAAACCAACAGCAATGACAGTAGGAGTAATTCAGCCTTCGCTGAAAACATGACATCAAACACACTCTGGTTTCCCTGAATCTGTTGCCTCCAGGTGTTAACACAGAATTAAGCATCCACAATTGCTGAAAGTCACCTGGGGCATGGTGGGTTTTGATCTTCTTCCCCTTCTTTTCTTCCCCTTCTTCTTTCCTTCTTTGATCTTCTTCCCCTTCTTTTCTTCCCCTTCCCCTTCTTTTCAATTTCTGCAATAAATTCAGACATGGACAGACACATTAAGCTGATTCCCCTACACACATAACAATCCACTGTCTAATCCTCACACAGGGACCTCAGGCTCCTCAGCATAAGAATAGGACACTGTGAGAGATATATTTCAGGAGGCCTGAGCGT
